# Dietary *Zanthoxylum bungeanum* Leaves Influence Meat Quality, Caecal Microbiota, Serum Metabolome and Muscle Transcriptome in Growing Rabbits

**DOI:** 10.3390/foods15132342

**Published:** 2026-07-02

**Authors:** Zhongqian Lu, Chunhui Deng, Zhengfeng Li, Shan Du, Xiaofeng Zhong, Qiuyang Liu, Yang Wang, Jingbo Liu, Jianfei Zhao

**Affiliations:** 1 College of Life Sciences and Agri-Forestry, Southwest University of Science and Technology, Mianyang 621010, China; 19913669769@163.com (Z.L.); 13458815351@163.com (C.D.); lizhengfeng1004@163.com (Z.L.); 15282228813@163.com (X.Z.); 15082212674@163.com (Q.L.); 19583607307@163.com (Y.W.); jingboliu@swust.edu.cn (J.L.); 2 Techlex Food Co., Ltd., Chengdu 610200, China; 18784004070@163.com; 3 Sichuan Provincial Engineering Research Center of Resource Utilization for Agricultural and Forestry Wastes, Chengdu Normal University, Chengdu 611130, China

**Keywords:** *Zanthoxylum bungeanum* leaves, fatty acid profile, gut microbiota, serum metabolomics, muscle transcriptome

## Abstract

This study evaluated the effects of dietary supplementation with 5% *Zanthoxylum* *bungeanum* leaf (ZBL) on growth performance, slaughter traits, meat quality, caecal microbiota, serum metabolome, and muscle transcriptome in rabbits. A total of 108 male New Zealand rabbits (60 days old) were randomly assigned to two groups (nine replicates/group; six rabbits/replicate) and fed either a basal diet (CON) or a diet in which 5% wheat bran was replaced with 5% ZBL for four weeks. Growth and slaughter performance did not differ (*p* > 0.05). ZBL reduced drip loss and cooking loss, enhanced antioxidant capacity, reduced specific saturated (C16:0), and unsaturated (C18:1 n-9 cis) fatty acids in leg muscle (*p* < 0.05), and the nutritional significance of these fatty acid changes remains unclear. ZBL also altered the levels of several volatile and non-volatile compounds in serum and muscle. It increased caecal abundance of *norank_f_Lachnospiraceae* and *Anaerofilum*, elevated serum metabolites (oleuropein, 3-coumaric acid), and upregulated meat quality-related genes (*NR3C2*, *PDZRN3*) in leg muscle (*p* < 0.05). Correlation analyses revealed that the observed changes in meat quality were closely associated with alterations in gut microbiota, serum metabolome, and muscle transcriptome. These findings suggest that dietary 5% ZBL does not compromise growth performance and is associated with changes in rabbit meat quality, which is associated with coordinated alterations in the gut microbiota, serum metabolome, and muscle transcriptome.

## 1. Introduction

Rabbit farming represents a significant segment of the global meat industry, with major production regions concentrated across Asia, Europe, the Americas, and Africa. Among these, China stands as the world’s largest producer of rabbit meat [1]. With its high protein, low fat, low cholesterol, and abundant unsaturated fatty acids and B vitamins, rabbit meat aligns with the increasing consumer demand for functional foods in modern diets [2,3]. Currently, the high incidence of stress and diseases in rabbits, largely attributable to existing farming practices and suboptimal environmental conditions, remains a significant challenge [4]. Therefore, the development of cost-effective novel feed ingredients that can enhance rabbits’ stress resistance and mitigate the detrimental effects of environmental stressors on rabbit meat quality is crucial for the sustainable advancement of the rabbit meat industry.

*Zanthoxylum bungeanum* (ZB), a species belonging to the Rutaceae family native to China, is commonly employed in both medicinal and culinary applications [5]. *Zanthoxylum bungeanum* leaf (ZBL), the primary by-product of ZB, possesses a unique aroma but is often used as fertilizer or discarded, leading to environmental pollution and a waste of natural resources [6]. ZBL is rich in proteins, lipids, phenolic compounds, flavonoids, and other essential nutrients, demonstrating antioxidant, antibacterial, and anti-inflammatory activities [7,8]. Appropriate dietary supplementation with ZBL has been shown to maintain production performance and enhance antioxidant capacity in broilers, effectively mitigating oxidative stress during rearing [9]. Furthermore, research by Cao et al. found that feeding ZBL significantly improves yolk color and flavor in eggs [10]. Thus, ZBL exhibits promising bioactive potential and represents a prospective feed supplement for improving the quality of livestock products. Given its rich phenolic and flavonoid content, ZBL may influence gut microbiota and host metabolism, potentially affecting muscle gene expression and meat quality through integrative multi-omics pathways. It is worth noting that *Zanthoxylum bungeanum* leaves contain anti-nutritional factors such as tannins, phytic acid, and alkaloids. Tannins can bind to dietary proteins and digestive enzymes, reducing protein digestibility and feed intake; phytic acid chelates minerals (e.g., Ca, Fe, Zn), impairing their bioavailability; and alkaloids may exert mild toxicity at high doses [11,12]. Therefore, the appropriate inclusion level of ZBL should be carefully considered in research and application contexts.

Based on this rationale, we hypothesized that dietary ZBL might affect rabbit meat quality through coordinated changes in gut microbiota, serum metabolome, and muscle transcriptome. Building on this rationale, the present study aimed to: (1) evaluate the effects of dietary 5% ZBL (replacing 5% wheat bran) on growth performance, slaughter traits, and meat quality in meat rabbits; (2) assess the impact of ZBL on caecal microbiota composition, serum metabolome, and leg muscle transcriptome; and (3) explore potential associations and correlative links among these multi-omics layers. This study provides a correlative framework for understanding ZBL as a functional feed additive in relation to meat quality changes. We seek to explore the functional value of ZBL in relation to mitigating environmental stress risks in animals and improving livestock product quality, thereby contributing to the advancement of meat rabbit farming and facilitating the resource utilization of agricultural by-products.

## 2. Materials and Methods

This experiment was carried out in the animal experimental base of Southwest University of Science and Technology. The animal experiments involved in this study have been approved by the Biological and Health Ethics Committee of Southwest University of Science and Technology (Approval No.: SM20250012).

### 2.1. Animals, Diets, and Treatment

A total of 108 healthy male New Zealand rabbits at 60 days of age, with similar body weights (1720 ± 100 g), were selected and randomly divided into two groups (CON group and ZBL group) with 9 replicates per group and 6 rabbits per replicate. The two groups were fed a basal diet and an experimental diet (ZBL replaces 5% bran), respectively.

Fresh *Zanthoxylum bungeanum* leaves were dried, ground, and passed through a 1 mm sieve (procured from Nanchong, China), and other feed ingredients were obtained from Mianyang Techlex Feed Industrial Co., Ltd. (Mianyang, China). The chemical composition and nutritional value of ZBL and wheat bran are presented in Table 1. It should be noted that only total phenolic and total flavonoid contents of ZBL were measured, without identification of individual compounds, as the primary focus was on integrated effects via multi-omics rather than comprehensive phytochemical profiling. The measured total contents serve as a sufficient proxy for the purposes of this trial. Future detailed profiling is warranted to identify specific bioactive constituents. The detailed diet composition and nutritional levels are presented in Table 2. The nutritional requirements were formulated according to the “Nutrient Requirements of Meat Rabbit” (NY/T 4049-2021) [13].

Feed was provided in the form of particles with a diameter of 3.2 mm and a length of 6~8 mm. All animals were maintained under identical housing management and environmental conditions. The experiment lasted for 4 weeks. During the experimental period, all rabbits had *ad libitum* access to feed and water.

### 2.2. Chemical Analysis

We determined the chemical composition of the experimental diets using standard methods. Specifically, we measured dry matter (DM; GB/T 6435–2014 [14]), crude protein (CP; GB/T 6432–2018 [15]), ether extract (EE; GB/T 6433–2006 [16]), crude fiber (CF; GB/T 6434–2022 [17]), crude ash (GB/T 6438–2007 [18]), calcium (Ca; GB/T 6436–2018 [19]), total phosphorus (TP; GB/T 6437–2018 [20]), gross energy (GE; ISO 9831:1998 [21]), neutral detergent fiber (NDF; GB/T 20806–2022 [22]) and acid detergent fiber (ADF; GB/T 20805–2022 [23]). Total flavonoids were assayed according to the SFDA’s Technical Guidelines for Health Foods (2020 Edition, Part 2, Section XV, Method II) [24]. Total polyphenols were measured following the group standard T/AHFIA 005–2018 [25].

### 2.3. Growth Performance

The body weight of each replicate group was recorded at the initiation of the trial as the initial body weight (IBW). Feed intake for each replicate group was measured accurately throughout the experimental period. At 20:00 on day 28, feed was removed, and final body weight (FBW) was recorded after a 12 h fasting period. Average daily gain (ADG), average daily feed intake (ADFI), and the feed-to-gain ratio (F/G) were calculated as follows:

ADG (g/d) = average weight gain (g)/number of feeding days (d);

ADFI (g/d) = average feed intake (g)/number of feeding days (d);

F/G = ADFI (g)/ADG (g).

### 2.4. Sample Collection and Processing

On trial day 29, the individual from each replicate with a body weight nearest to the replicate mean was selected. Thus, a total of nine rabbits per dietary treatment (one from each of nine replicates) were selected for slaughter and subsequent analyses. After body weight measurement, euthanasia was performed via intravenous air embolism (10 mL air) through the marginal ear vein, after deep anesthesia with sodium pentobarbital (30 mg/kg, i.v.). This procedure complied with both the Chinese national standard GB/T 35823–2018 [26] and the AVMA Guidelines for the Euthanasia of Animals (2020 Edition) [27]. The heart, liver, kidneys, spleen, thymus, sacculus rotundus, and left leg muscle were completely excised and weighed. The left leg biceps femoris muscle was excised and stored at 4 °C for subsequent measurement of muscle pH and meat color at 45 min and 24 h post-mortem. The left leg quadriceps femoris muscle was separated and used to determine cooking loss and shear force. Dressed weights were recorded, including both semi-eviscerated and full-eviscerated carcass weights. Blood samples were collected and allowed to stand at 37 °C for 30 min, followed by centrifugation at 1300× *g* for 15 min. The upper serum layer was collected, aliquoted, and stored at −80 °C until analysis. A sample of appropriate size was collected from the biceps femoris of the right hind limb for drip loss measurement. Additionally, a muscle tissue sample from the right quadriceps femoris was obtained, placed in a cryovial, and subsequently stored at −80 °C in a freezer until further analysis. Additionally, caecal contents were preserved at −80 °C for subsequent 16S rRNA sequencing.

### 2.5. Slaughter Performance and Relative Organ Weight

Slaughter performance and relative organ weights were determined according to the protocols established by Zhang et al. [28]. For meat rabbits, the full-eviscerated carcass weight refers to the weight after slaughter following removal of blood, fur, head, tail, forelimbs (distal to the carpal joint), hindlimbs (distal to the tarsal joint), and all internal organs (including abdominal fat). The semi-eviscerated carcass weight is defined as the full-eviscerated carcass weight plus the retained heart, liver, kidney, spleen, thymus, and sacculus rotundus. The following formulas were used to calculate the dressing percentages and relative organ weight:

Full-eviscerated dressing percentage (%) = [Full-eviscerated carcass weight (g)/Live weight before slaughter (g)] × 100;

Semi-eviscerated dressing percentage (%) = [Semi-eviscerated carcass weight (g)/Live weight before slaughter (g)] × 100;

Leg muscle rate (%) = [Left leg muscle weight (g) × 2/Full-eviscerated carcass weight (g)] × 100;

Relative organ weight (g/kg) = [Fresh organ weight (g)/Live weight before slaughter (kg)].

### 2.6. Leg Muscle Quality

The pH of the left biceps femoris muscle was measured at 45 min and 24 h post-mortem using a pH-STAR portable pH meter (MATTHAUS, Pottenmes, Germany). Calibration was performed with standard buffers (pH 4.0 and 7.0) before measurements. Values are presented as the mean of three replicates. Meat color (at 45 min and 24 h post-mortem) was measured using a CM 2500c spectrophotometer (Konica Minolta, Tokyo, Japan) with an 8 mm aperture, D65 illuminant, and 2° observer. The specific muscle used was the biceps femoris. Measurements were performed immediately after cutting the muscle surface, without a blooming period. Between the two measurement time points (45 min and 24 h post-mortem), the meat samples were stored at 4 °C. Parameters included lightness (L*), redness (a*), and yellowness (b*). Calibration was conducted against a standard white tile (L* 89.2, a* 0.921, b* 0.783). Three points on the cross-section of the left biceps femoris were assessed, and the average value was calculated.

A sample of approximately 20–25 g (approximately 3 cm in thickness) was taken from the right biceps femoris muscle, suspended in a sealed container, and stored at 4 °C for 24 h before being weighed. Drip loss was calculated as (initial weight − final weight)/initial weight × 100%. A tissue sample weighing approximately 15 g (approximately 2 cm in thickness) was harvested from the right quadriceps femoris muscle. The specimen was immediately placed into a vacuum-sealed bag, from which the air was completely evacuated. Subsequently, the sealed sample was subjected to heat treatment in a water bath set at 90 °C for 30 min. After cooking, the sample was allowed to cool to room temperature under ambient conditions. Once cooled, the final weight of the sample was recorded. Cooking loss was calculated as (raw weight − cooked weight)/raw weight × 100%.

From the aforementioned cooked sample, a 30 mm long cylindrical core (10 mm × 10 mm in cross-section, cut parallel to the muscle fibers) was prepared. The specimen was then sheared at a constant speed of 200 mm/min using a texture analyzer (TA-XT2, Stable Micro Systems, Godalming, UK). The maximum shear force was recorded and expressed in newtons (N).

### 2.7. Antioxidant Indicators in Leg Muscle

A 10% (*w*/*v*) tissue homogenate was prepared from the right quadriceps femoris muscle by homogenizing the sample in normal saline at a ratio of 1:9 (*w*/*v*). The following parameters were measured: total antioxidant capacity (T-AOC, kit A015-1-2), total superoxide dismutase (T-SOD, A001-1-2), catalase (CAT, A007-1-1), and glutathione peroxidase (GSH-PX, A005-1-2) activities, as well as malondialdehyde (MDA, A003-1-1) content. All assays were conducted following the manufacturer’s protocols using commercial kits sourced from Nanjing Jiancheng Bioengineering Institute (Nanjing, China).

### 2.8. Fatty Acid Profile in Leg Muscles

This analysis was performed according to the method described by Yan et al. [29] and Zhu et al. [30] for the quantification of 51 medium- and long-chain fatty acids in muscle tissue. Approximately 50 mg of the right quadriceps femoris muscle was thawed on ice and homogenized with pre-cooled beads using a bead mill at 30 Hz for 3 min. Then, 1 mL of 70% methanol was added, followed by vortexing for 5 min and centrifugation at 12,000 rpm for 10 min at 4 °C. A 400 µL aliquot of the supernatant was collected and stored at −20 °C overnight. After a second centrifugation step, the resulting supernatant was subjected to instrumental analysis. Fatty acids were extracted, methylated, and analyzed by GC-MS following established protocols [29]. Separation used an Agilent DB-FAST FAME column (30 m × 250 μm × 0.25 μm, Agilent Technologies, Santa Clara, CA, USA) with high-purity helium (1.0 mL/min). The injector was set at 260 °C, with 1 µL injection, 10:1 split ratio, and 9.9 min solvent delay. The oven program was 80 °C, ramped to 168 °C at 70 °C/min, then to 175 °C at 1.5 °C/min, and finally to 230 °C at 13 °C/min (held 4.5 min). Mass spectra were acquired in SIM mode (EI, 70 eV) with the ion source at 230 °C, quadrupole at 150 °C, and transfer line at 240 °C. Identification used retention time matching and mass spectral comparison with authentic standards (Sigma-Aldrich, St. Louis, MO, USA); quantification used isotope-labeled internal standards. System stability was validated (RSD < 15% for all targets). LOD and LOQ were set at S/N 3:1 and 10:1; fatty acids below LOQ were excluded. Values represent only predefined target panel fatty acids, not total lipid content.

### 2.9. Metabolomic Analysis of Flavor-Related Metabolites in Leg Muscles

Frozen right quadriceps femoris muscle samples (−80 °C) were pulverized under liquid nitrogen to obtain a homogeneous powder. A 50 mg aliquot of the powder was transferred into a centrifuge tube containing 0.5 mL of a pre-cooled methanol–water mixture (4:1, *v*/*v*) spiked with ribitol (0.02 mg/mL) as an internal standard. Following thorough vortex mixing, the suspension was subjected to low-temperature ultrasonic-assisted extraction. The extract was then centrifuged at 12,000× *g* for 15 min at 4 °C, and the resulting supernatant was evaporated to dryness under a gentle stream of nitrogen. The dried residue was reconstituted in 10% methanol, centrifuged again, and the clear supernatant was analyzed by gas chromatography–mass spectrometry (GC–MS).

Chromatographic separation was achieved on a GC system equipped with a TG-5SILMS capillary column (30 m × 0.25 mm × 0.25 µm, Thermo 26096-1420, Thermo Fisher Scientific, Waltham, MA, USA). High-purity helium served as the carrier gas, and a temperature gradient program was applied to optimize resolution. Mass spectra were acquired using electron impact ionization (70 eV) in full-scan mode over a mass range of *m/z* 35–500. Metabolites—including amino acids, organic acids, sugars, fatty acids, and their derivatives—were identified by comparing the obtained mass spectra against the NIST-2023 database, with retention index data used for additional confirmation.

### 2.10. 16S rRNA Microbiome Sequencing

Total genomic DNA was isolated from the cecal microbial community using the E.Z.N.A.^®^ Stool DNA Kit (Omega Bio-tek, Norcross, GA, USA), following the protocol supplied by the manufacturer. The quality, concentration, and purity of the extracted DNA were subsequently assessed. Using the purified DNA as a template, the V3–V4 hypervariable region of the bacterial 16S rRNA gene was amplified via PCR with the barcoded forward primer 338F (5′-ACTCCTACGGGAGGCAGCAG-3′) and reverse primer 806R (5′-GGACTACHVGGGTWTCTAAT-3′). Amplified products were then purified, quantified, and employed to construct sequencing libraries using the NEXTFLEX Rapid DNA-Seq Kit. Sequencing was carried out on an Illumina NextSeq 2000 platform at Shanghai Majorbio Bio-pharm Technology Co., Ltd. (Shanghai, China). All downstream bioinformatic analyses, including alpha diversity indices (Chao1, Shannon, Simpson), principal coordinate analysis (PCoA), microbial community composition profiling, and linear discriminant analysis effect size (LEfSe), were performed on the Majorbio Cloud Platform (https://cloud.majorbio.com).

### 2.11. Serum Metabolomics Analysis

An aliquot of serum (100 μL) was combined with an extraction solvent composed of acetonitrile and methanol (1:1, *v*/*v*) and briefly vortex-mixed for 30 s. Metabolite extraction was carried out using low-temperature ultrasonication at 5 °C with a frequency of 40 kHz for 30 min. The mixture was then left to stand at −20 °C for 30 min, followed by centrifugation at 4 °C and 13,000× *g* for 15 min. The resulting supernatant was collected, evaporated to dryness under a stream of nitrogen gas, and redissolved in 100 μL of a reconstitution buffer consisting of acetonitrile and water (1:1, *v*/*v*). The reconstituted sample was subjected to a second round of low-temperature ultrasonication (5 °C, 40 kHz, 5 min) and centrifugation (4 °C, 13,000× *g*, 10 min). The final supernatant was transferred for LC-MS analysis.

LC-MS analysis was conducted using an ultra-high-performance liquid chromatography system coupled to a Fourier transform mass spectrometer (UHPLC-Orbitrap Exploris 240, Thermo Fisher Scientific, Waltham, MA, USA), operated at Shanghai Majorbio Bio-pharm Technology Co., Ltd. (Shanghai, China). Chromatographic separation was performed on an HSS T3 column (100 mm × 2.1 mm inner diameter, 1.8 μm particle size; Waters, Milford, MA, USA) maintained at 40 °C. The mobile phase consisted of solvent A (water/acetonitrile, 95:5, *v*/*v*) containing 0.1% formic acid, and solvent B (acetonitrile/isopropanol/water, 47.5:47.5:5, *v*/*v*/*v*) also containing 0.1% formic acid. The flow rate was set to 0.40 mL/min. Mass spectra were acquired in both positive and negative electrospray ionization modes over a mass-to-charge (*m*/*z*) range of 70–1050. The ion spray voltages were 3500 V in positive mode and −3000 V in negative mode. The sheath gas and auxiliary gas pressures were set to 50 arbitrary units (arb) and 13 arb, respectively, with an ion transfer tube temperature of 450 °C. Collision energies were applied in a stepped manner at 20, 40, and 60 V.

### 2.12. Leg Muscle Transcriptomic Analysis

Total RNA was extracted from the right quadriceps femoris muscle using QI-Azol Lysis Reagent (Qiagen, Hilden, Germany) in accordance with the manufacturer’s instructions. RNA concentration and purity were evaluated using a Nanodrop 2000 spectrophotometer (Thermo Fisher Scientific, Waltham, MA, USA), whereas RNA integrity was examined via agarose gel electrophoresis. The RNA Quality Number (RQN) was determined using an Agilent 5300 Bioanalyzer (Agilent, Santa Clara, CA, USA). Only RNA specimens meeting the following quality thresholds were selected for subsequent library construction: total RNA amount ≥ 1 μg, concentration ≥ 30 ng/μL, RQN > 6.5, and an OD260/280 ratio ranging from 1.8 to 2.2.

Polyadenylated mRNA was captured from total RNA using oligo(dT)-conjugated magnetic beads. The isolated mRNA was subsequently fragmented into fragments of approximately 300 bp using a fragmentation buffer. First-strand complementary DNA (cDNA) was synthesized from the fragmented mRNA using random primers and reverse transcriptase, followed by second-strand synthesis to produce double-stranded cDNA. The resulting cDNA underwent end-repair using an End Repair Mix to generate blunt ends, after which a single adenine (A) overhang was added to the 3′ ends to enable adapter ligation. Following adapter ligation, the products were purified and subjected to size selection. The selected cDNA fragments were then amplified via PCR, and the constructed library was purified. High-throughput sequencing was ultimately conducted on the NovaSeq X Plus platform (Illumina, San Diego, CA, USA).

### 2.13. qRT-PCR Analysis

To confirm the reproducibility of the differentially expressed genes (DEGs) identified from the RNA-seq data in this study, seven selected DEGs were validated by qRT-PCR using the same RNA samples as those used for RNA-seq. The corresponding primer sequences are provided in Table 3, and all primers were designed and cross-checked against the NCBI database.

### 2.14. Statistical Analysis

Phenotypic data were analyzed by independent samples *t*-test using SPSS Statistics 27.0 and are presented as mean ± standard error (SE). Statistical significance was defined as *p* < 0.05, with *p* < 0.01 considered highly significant.

For caecal microbiome analysis, alpha diversity indices were compared using the Wilcoxon rank-sum test, and *p*-values were adjusted for multiple testing using the false discovery rate (FDR) method. Beta diversity was assessed by PCoA, and group differences were tested using Adonis. Differential microbial features were identified with LEfSe under the criteria of LDA score > 2 and *p* < 0.05. For serum metabolomics, principal component analysis (PCA) was conducted, and group separation was evaluated by Adonis; metabolite differences between groups were analyzed via *t*-tests. KEGG pathway enrichment analysis for differential metabolites was performed with Benjamini–Hochberg (BH) correction. In muscle transcriptome analysis, differential gene expression was determined using DESeq2, with *p*-values adjusted via the BH procedure. Gene Ontology (GO) and KEGG pathway enrichment analyses were also corrected for multiple comparisons using the BH method.

## 3. Results

### 3.1. Growth and Slaughter Performance

Table 4 presents the growth performance results. Dietary ZBL supplementation did not significantly affect initial body weight, initial weight, final weight, ADG, ADFI, or F/G (*p* > 0.05). Table 5 shows the slaughter performance and relative organ weight. No significant differences were observed between the two groups in semi-eviscerated dressing percentage, eviscerated dressing percentage, leg muscle yield, or the indices of heart, liver, spleen, or sacculus rotundus (*p* > 0.05).

### 3.2. Effect of ZBL on Leg Muscle Meat Quality in Meat Rabbits

Table 6 summarizes the leg muscle quality parameters. Compared with the CON group, the pH, meat color (L*, a*, b*), and shear force of leg muscle in meat rabbits from the ZBL group showed no significant changes at 45 min and 24 h post-slaughter (*p* > 0.05). However, the drip loss and cooking loss were significantly reduced in the ZBL group (*p* < 0.05). Table 7 presents the antioxidant capacity of leg muscle. Dietary ZBL also significantly enhanced the activities of GSH-Px (*p* < 0.01), T-AOC, CAT, and T-SOD (*p* < 0.05) in leg muscle, while decreasing MDA content (*p* < 0.05).

Table 8 shows the fatty acid composition of leg muscle. ZBL supplementation significantly reduced total saturated fatty acid (SFA) (*p* < 0.01) and total unsaturated fatty acid (UFA) contents (*p* < 0.05), primarily reflected by a marked reduction in monounsaturated fatty acids (MUFA) (*p* < 0.05). Among the SFAs, significant decreases were observed in C12:0, C14:0, C15:0, and C20:0 (*p* < 0.05), while C16:0 was highly significantly reduced (*p* < 0.01). For UFAs, significant reductions were detected in C14:1 n-5, C16:1 n-7, C18:1 n-9 cis, C18:3 n-6, and C18:3 n-3 (*p* < 0.05). No significant effects were observed on polyunsaturated fatty acids (PUFA), n-3/n-6 PUFA, atherogenic index (AI) or thrombogenic index (TI) (*p* > 0.05).

**Figure 1:**
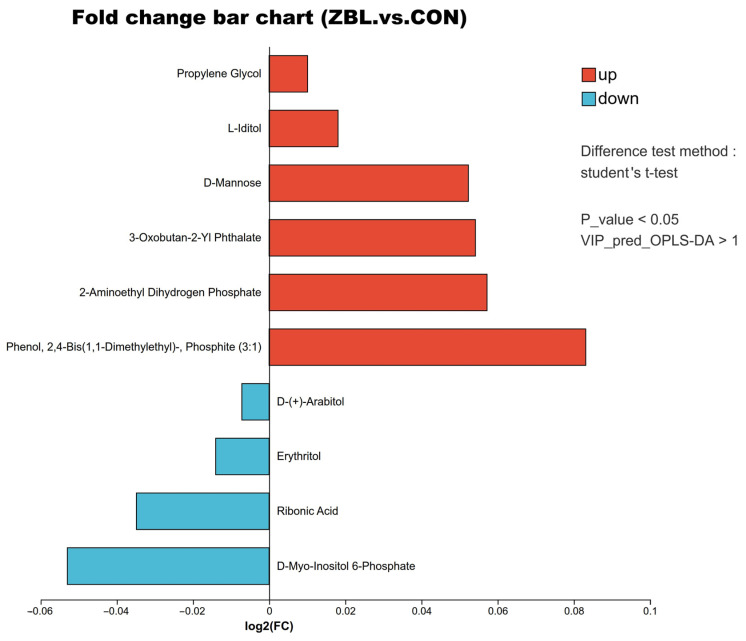
Differential taste-related metabolites in the leg muscle of meat rabbits (ZBL vs. CON). The fold change bar chart displays the log2 fold change (log2FC) of each metabolite between ZBL and CON, with corresponding *p*-values. Red indicates metabolites with increased abundance in the ZBL group compared to CON, while blue indicates decreased abundance. Only metabolites with significant differences (*p* < 0.05) are shown. (*n* = 9 per group).

Figure 1 illustrates the changes in flavor-related metabolites in leg muscle. Dietary ZBL significantly increased the levels of propylene glycol, L-iditol, D-mannose, 3-oxobutan-2-yl phthalate, 2-aminoethyl dihydrogen phosphate, and phenol, 2,4-bis(1,1-dimethylethyl)-, phosphite (3:1) (*p* < 0.05). Conversely, the levels of D-(+)-arabitol, erythritol, ribonic acid, D-myo-inositol 6-phosphate, and a tetraoxa-trisilaheptane derivative were significantly decreased (*p* < 0.05).

### 3.3. Caecal Microbiota Composition

**Figure 2:**
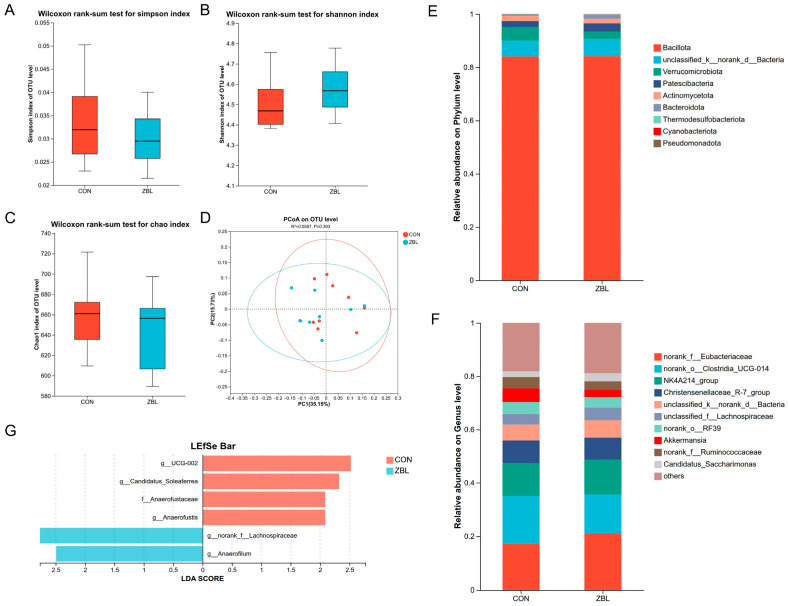
Diversity and compositional abundance of the caecal microbiota. (**A**) Simpson index; (**B**) Shannon index; (**C**) Chao index; (**D**) PCoA analysis; (**E**) top 10 phyla by relative abundance; (**F**) top 10 genera by relative abundance; (**G**) LEfSe analysis. (*n* = 9 per group).

Figure 2 displays the caecal microbiota analysis. Dietary supplementation with *Zanthoxylum bungeanum* leaves did not significantly affect the alpha diversity (Simpson, Shannon, and Chao indices) or beta diversity (PCoA) of caecal microbiota in meat rabbits (*p* > 0.05; Figure 2A–D). Taxonomic composition analysis revealed that at the phylum level, the dominant phyla were *Bacillota*, *unclassified_k__norank_d__Bacteria*, *Verrucomicrobiota*, *Patescibacteria*, *Actinomycetota*, *Bacteroidota*, *Thermodesulfobacteriota*, *Cyanobacteriota*, and *Pseudomonadota* (Figure 2E). At the genus level, the predominant genera included *norank_f__Eubacteriaceae*, *norank_o__Clostridia_UCG-014*, *NK4A214_group*, *Christensenellaceae_R-7_group*, *unclassified_k__norank_d__Bacteria*, *unclassified_f__Lachnospiraceae*, *norank_o__RF39*, *Akkermansia*, *norank_f__Ruminococcaceae*, *Candidatus_Saccharimonas*, and others (Figure 2F). LEfSe analysis indicated that the CON group was significantly enriched in the genera *UCG-002*, *Candidatus_Soleaferrea*, and *Anaerofustis*, whereas the ZBL group showed significant enrichment in *norank_f__Lachnospiraceae* and *Anaerofilum* (Figure 2G).

### 3.4. Serum Metabolome

**Figure 3:**
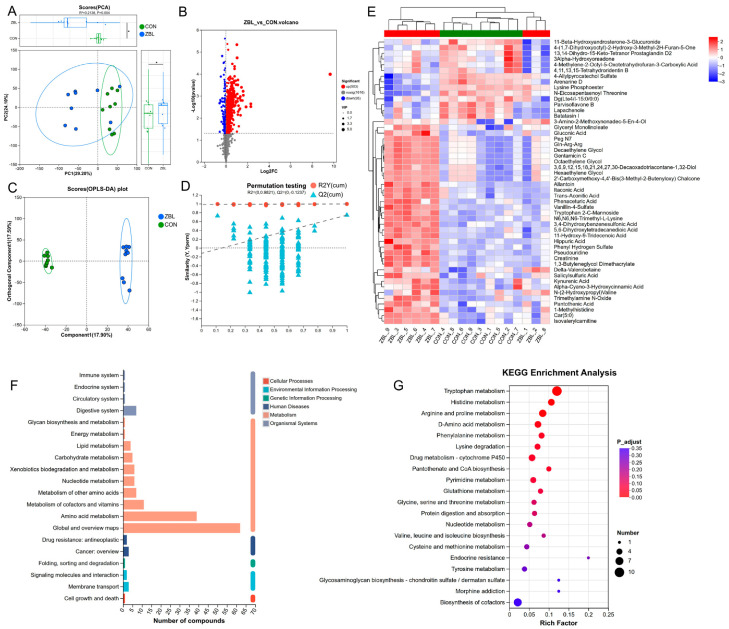
Serum metabolite composition analysis and KEGG functional pathway analysis. (**A**) PCA score plot. (**B**) Volcano plot of differential metabolites; (**C**) OPLS-DA score plot; (**D**) permutation test scatter plot for the OPLS-DA model; (**E**) clustering heatmap of differential metabolites (top 50 in abundance); (**F**) bar plot of KEGG level-2 functional pathway statistics for differential metabolites (top 20 in abundance); (**G**) bubble chart of KEGG functional pathway enrichment for differential metabolites (top 20 by *p*-adjust). (*n* = 9 per group).* *p* < 0.05.

Serum metabolomics analysis results are shown in Figure 3. Figure 3A shows a significant separation between the CON and ZBL groups by PCA (*p* = 0.004), which was further supported by the clear distinction observed in the OPLS-DA results (Figure 3C,D). A total of 2214 metabolites were detected, with 503 significantly upregulated and 95 downregulated in the ZBL group (Figure 3B). As illustrated in the clustering heatmap, the differentially abundant metabolites with higher relative levels were primarily steroids, lipids, amino acid derivatives, organic acids, and other bioactive molecules (Figure 3E). KEGG functional classification indicated that these differential metabolites were predominantly associated with Metabolism, mainly distributed within Global and overview maps, Amino acid metabolism, Metabolism of cofactors and vitamins, and Metabolism of other amino acids. Additionally, metabolites related to the Digestive system constituted a notable proportion (Figure 3F). Enrichment analysis of KEGG pathways identified significant enrichment in Tryptophan metabolism, Histidine metabolism, Arginine and proline metabolism, D-Amino acid metabolism, Phenylalanine metabolism, Lysine degradation, Drug metabolism-cytochrome P450, and Pantothenate and CoA biosynthesis (*p*-adjust < 0.05, Figure 3G).

### 3.5. Leg Muscle Transcriptome

**Figure 4:**
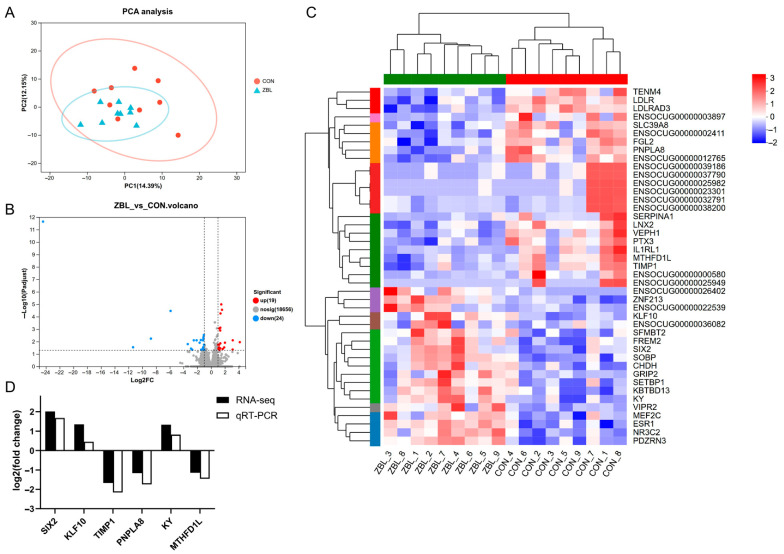
Analysis of gene expression in muscle tissue. (**A**) Principal component analysis (PCA) of samples; (**B**) volcano plot of differentially expressed genes; (**C**) heatmap with hierarchical clustering of differentially expressed genes (unnamed genes are labeled by gene_id); (**D**) qRT-PCR validation of differentially expressed genes in the leg muscles of meat rabbits. (*n* = 9 per group).

**Figure 5:**
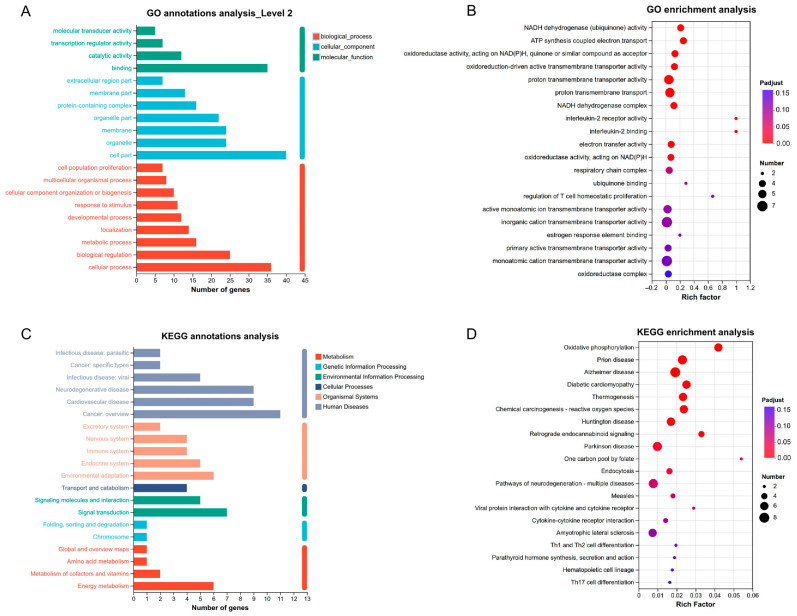
Functional enrichment analysis of differentially expressed genes in muscle tissue: Gene Ontology (GO) and KEGG pathway analyses. (**A**) Bar plot of top 20 abundant GO secondary terms. (**B**) Bubble plot of the top 20 significantly enriched GO terms (ranked by *p*-adjust). (**C**) Bar plot of top 20 abundant KEGG secondary pathways. (**D**) Bubble plot of the top 20 significantly enriched KEGG pathways (ranked by *p*-adjust). (*n* = 9 per group).

Figure 4 and Figure 5 present the transcriptomic analysis of leg muscle. PCA did not show a clear separation between the CON and ZBL groups within the confidence ellipses (Figure 4A). Dietary ZBL supplementation significantly upregulated 19 genes and downregulated 24 genes in rabbit leg muscle (Figure 4B). The differentially expressed genes (DEGs) encoded transcriptional regulators, signal transduction receptors, metabolic enzymes, extracellular matrix proteins, and ubiquitin ligases. Among these, *MEF2C*, *ESR1*, *NR3C2*, *KBTBD13*, *KY*, *PDZRN3*, and *VEPH1* are potentially associated with meat quality traits (Figure 4C). qRT-PCR validation of six selected genes (*SIX2*, *KLF10*, *TIMP1*, *PNPLA8*, *KY*, and *MTHFD1L*) confirmed the RNA-seq expression trends (Figure 4D).

GO enrichment analysis (Figure 5A,B) showed that the DEGs were significantly enriched in molecular functions such as NADH dehydrogenase (ubiquinone) activity, oxidoreductase activity acting on NAD(P)H, and proton transmembrane transporter activity, as well as biological processes, including proton transmembrane transport and ATP synthesis coupled electron transport (Figure 5B). KEGG pathway analysis (Figure 5C,D) revealed that the DEGs were significantly enriched in oxidative phosphorylation, thermogenesis, and chemical carcinogenesis-reactive oxygen species pathways (*p*-adjust < 0.05; Figure 5D).

### 3.6. Multi-Omics Correlation Analysis

**Figure 6:**
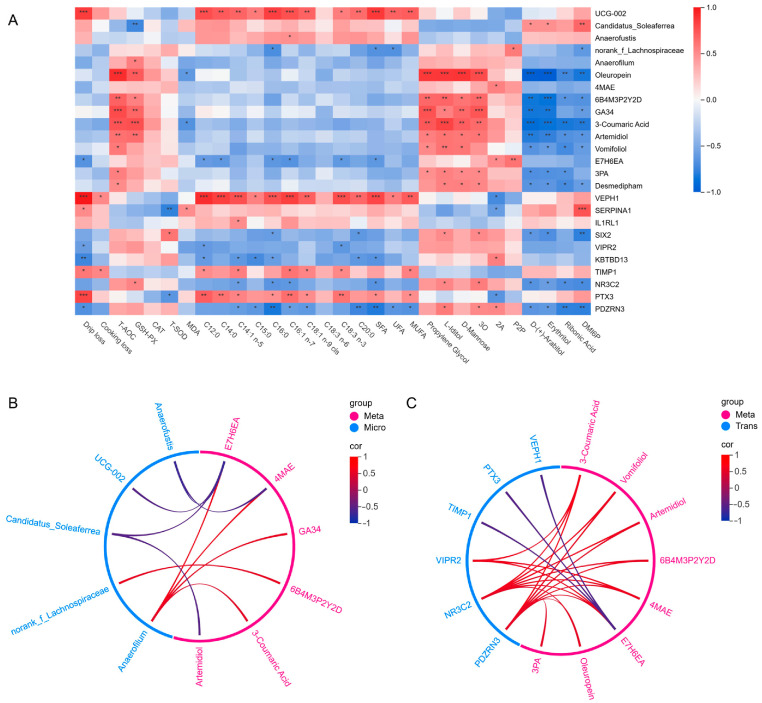
Multi-omics correlation analysis. (**A**) Heatmap depicting Spearman’s rank correlations between differential caecal microorganisms, serum metabolites (top ten metabolites ranked by *p*-value and fold change), and muscle differentially expressed genes (DEGs, top ten genes ranked by *p*-value and fold change) in relation to differential phenotypes. * *p* < 0.05, ** *p* < 0.01, *** *p* < 0.001. (**B**) Chord diagram illustrating significant Spearman correlations (*p* < 0.05) between differential caecal microorganisms and serum metabolites. (**C**) Chord diagram showing significant Spearman correlations (*p* < 0.05) between differential serum metabolites and muscle DEGs. 4MAE: 4-Methyl-Alpha-Ethyltryptamine; 6B4M3P2Y2D: 6-Benzyl-4-Methyl-3-Propan-2-Ylmorpholine-2,5-Dione; GA34: Gibberellin A34-Catabolite; E7H6EA: (E)-7-(4-Methoxy-5-Methyl-6-Oxopyran-2-Yl)Hept-6-Enoic Acid; 3PA: 3-Phenylpropionic Acid; 3O: 3-Oxobutan-2-YI Phthalate; 2A: 2-Aminoethyl Dihydrogen Phosphate; P2P: Phenol, 2,4-Bis(1,1-Dimethylethyl)-, Phosphite (3:1); DMI6P: D-Myo-Inositol 6-Phosphate.

Figure 6 illustrates the multi-omics correlation analysis. Muscle antioxidant capacity and flavor-related metabolites were strongly correlated with serum metabolites, whereas changes in drip loss and fatty acid composition were more closely associated with muscle gene expression (Figure 6A). The abundance of the caecal microbe UCG-002 showed a significant positive correlation with muscle fatty acid content (Figure 6A). Differential microbial taxa were predominantly correlated with serum metabolites, including (E)-7-(4-methoxy-5-methyl-6-oxopyran-2-yl)hept-6-enoic acid, 4-methyl-alpha-ethyltryptamine, gibberellin A34-catabolite, 6-benzyl-4-methyl-3-propan-2-ylmorpholine-2,5-dione, 3-coumaric acid, and artemidiol (Figure 6B). In turn, these metabolites were significantly correlated with the expression of several muscle genes, including *VEPH1*, *PTX3*, *TIMP1*, *VIPR2*, *NR3C2*, and *PDZRN3* (Figure 6C).

## 4. Discussion

ZBL represents a plant-based, unconventional feed resource with substantial application potential, owing to its diverse nutritional components and bioactive substances [31,32]. Previous studies have demonstrated that ZBL can, to some extent, enhance the quality of poultry eggs and meat [10,33]. However, its application in feed is limited due to the presence of anti-nutritional factors such as alkylamides [12,33]. In the present study, dietary supplementation with 5% ZBL did not compromise growth performance or slaughter traits in meat rabbits, indicating that at this inclusion level, any adverse effects were negligible. This safety profile aligns with previous reports in broilers (Hu et al., 2024 [9]). More importantly, ZBL inclusion was associated with improved meat quality traits, including enhanced water-holding capacity (reduced drip and cooking loss), increased muscle antioxidant enzyme activities, decreased MDA content, and altered fatty acid and flavor-related metabolite profiles. Based on multi-omics correlation analysis, we hypothesize that these benefits are associated with coordinated changes in gut microbiota, serum metabolites, and muscle gene expression.

The improvement in water-holding capacity was closely correlated with enhanced antioxidant status. Oxidative stress is known to disrupt sarcolemmal integrity, leading to post-mortem fluid loss [34]. ZBL inclusion was associated with elevated activities of key antioxidant enzymes and reduced lipid peroxidation. This association may reflect two non-exclusive pathways: direct scavenging of reactive oxygen species by ZBL-derived polyphenols [7,8], and indirect modulation of the gut microbiota. Our 16S rRNA data showed an increased abundance of *norank_f_Lachnospiraceae* and *Anaerofilum* in ZBL-fed rabbits. Members of *Lachnospiraceae* are known producers of short-chain fatty acids (SCFAs), particularly butyrate [35,36], and butyrate has been reported to activate the Nrf2 signaling pathway, which enhances systemic antioxidant capacity [37]. In support of this, muscle transcriptomic analysis revealed that ZBL inclusion was associated with upregulation of oxidative phosphorylation-related pathways and genes such as *NR3C2* and *PDZRN3*, which are involved in electrolyte balance and myofiber integrity [38,39]. Although these correlations are consistent, causality cannot be inferred; for instance, whether SCFA levels directly increase and whether Nrf2 is activated in muscle remains to be tested experimentally.

ZBL inclusion was also associated with reduced levels of specific saturated (C14:0, C16:0) and monounsaturated fatty acids (C18:1 n-9 cis) in leg muscle, which is consistent with the reported lipid-lowering bioactivity of ZBL [40]. Two correlative observations are noteworthy. First, serum metabolomics identified elevated oleuropein and 3-phenylpropionic acid—the latter is a microbial metabolite that has been associated with beige adipogenesis and energy expenditure [41]. Second, the muscle transcriptome showed altered expression of *PDZRN3*, a gene implicated in satellite cell differentiation and lipid metabolism [42]. Previous studies have shown that *PDZRN3* negatively regulates adipocyte differentiation and is essential for the commitment of mesenchymal progenitor cells to myogenic differentiation [43]. Collectively, these findings suggest that ZBL may influence intramuscular fat deposition through pathways involving direct absorption of phenolic compounds and microbiota-derived metabolites, although further investigation is warranted to confirm this hypothesis. The reduction in SFAs is generally considered nutritionally favorable [44]. However, the concurrent decrease in C18:1 n-9 cis (MUFA) and several n-3 fatty acids may partially offset this advantage. Notably, although ZBL treatment reduced the levels of certain fatty acids in rabbit meat, it did not significantly affect the Σ n-6/Σ n-3 ratio, indicating that the overall fatty acid composition of the meat remained largely unaltered. Therefore, the net nutritional significance of these simultaneous changes remains unclear, and definitive conclusions regarding the nutritional superiority of ZBL-supplemented meat cannot be drawn from these data alone. We acknowledge that leg muscle proximate composition was not determined in this study. Despite the uncertainty in fatty acid interpretation, ZBL supplementation consistently improved other meat quality attributes, including reduced drip loss and cooking loss, as well as enhanced antioxidant capacity. These improvements, together with the unaltered growth performance, support the potential of ZBL as a practical partial replacement for wheat bran in rabbit diets. In addition to fatty acid changes, ZBL inclusion was associated with changes in flavor-related metabolites, including increased D-mannose (a Maillard reaction precursor) and decreased certain sugar alcohols. However, it is important to emphasize that changes in metabolite levels do not necessarily translate to enhanced palatability or consumer preference. Furthermore, no sensory evaluation was performed in this study. Therefore, whether these metabolic changes result in perceptible differences in flavor or consumer acceptance requires direct sensory testing in future studies.

Integrating the multi-omics correlation analyses, we propose a working hypothesis that links the observed changes across gut microbiota, serum metabolites, and muscle transcriptome. Specifically, dietary ZBL was associated with a higher abundance of *norank_f_Lachnospiraceae* and *Anaerofilum*. These taxa have been implicated in SCFA production, although we did not directly measure SCFAs in this study. The altered microbial composition correlated with changes in serum metabolites, including 3-coumaric acid, 4-methyl-alpha-ethyltryptamine, oleuropein, and 3-phenylpropionic acid. Some of these metabolites (3-phenylpropionic acid) are known to be produced by *Lachnospiraceae* (Li et al., 2026 [41]), while others (oleuropein) are likely derived directly from ZBL. In turn, several of these serum metabolites showed positive correlations with the expression of muscle genes such as *NR3C2* and *PDZRN3*, which we found to be associated with water-holding capacity and fatty acid composition. This “gut microbiota–serum metabolome–muscle transcriptome” axis represents a plausible integrative model. However, it is critical to emphasize that the current evidence is entirely correlative. The specific bacterial taxa identified account for only a small fraction of the microbial community, and their functional contribution to the production of the measured metabolites has not been directly confirmed. Moreover, the transcriptomic changes involve a modest number of genes, and the functional relevance of *NR3C2* and *PDZRN3* in rabbit muscle awaits experimental validation via gene knockdown or overexpression in myocytes. Additionally, we did not measure SCFAs directly, nor did we perform functional assays for Nrf2 activation or lipogenic enzyme activities. Therefore, while the correlative framework is informative, it does not establish directionality or causation.

Several other limitations should be acknowledged. First, the study used a single inclusion level of ZBL without a dose–response design, precluding determination of an optimal or threshold effect. Second, the 4-week feeding period may not capture long-term effects or adaptation of the gut microbiota. Third, the chemical composition of ZBL, particularly the content and profile of individual polyphenols, was not characterized, limiting our ability to link specific compounds to the observed associations. Future studies should address these gaps by combining metabolomic and transcriptomic data with targeted biochemical assays and in vitro models using myotubes or primary rabbit muscle cells to test causality. Furthermore, a sensory panel would clarify the practical implications of the altered flavor-related metabolites. Despite these limitations, the present study provides a comprehensive multi-omics landscape that can guide future hypothesis-driven research on ZBL as a functional feed ingredient.

## 5. Conclusions

Dietary inclusion of 5% ZBL (replacing an equal proportion of wheat bran) did not adversely affect growth performance or slaughter traits in meat rabbits. It was associated with improved water-holding capacity and antioxidant status of leg muscle, as well as altered fatty acid and flavor-related metabolite profiles. Multi-omics correlation analysis revealed coordinated associations among increased caecal abundance of *norank_f_Lachnospiraceae* and *Anaerofilum*, shifts in serum metabolites, including oleuropein and 3-coumaric acid, and changes in muscle gene expression such as *NR3C2* and *PDZRN3*. These findings provide a multi-omics correlative landscape linking dietary ZBL to meat quality traits in rabbits, though the biological significance of these observed associations requires further investigation.

## Figures and Tables

**Table 1:** Proximate composition and bioactive compound content of *Zanthoxylum bungeanum* leaf and wheat bran (on an air-dry basis, %).

Items ^1^	*Zanthoxylum bungeanum* Leaf	Wheat Bran
Proximate Composition		
DM	88.85	87.00
CP	15.00	14.30
Ash	10.30	4.80
EE	5.20	4.00
CF	17.00	6.80
NDF	27.20	41.30
ADF	18.90	11.90
Ca	2.78	0.10
TP	0.23	0.93
GE (MJ/kg)	15.99	17.53
Bioactive Compounds		
Total flavone (g/kg)	53.31	-
Total polyphenol (g/kg)	43.37	-

^1^ Abbreviations: DM, dry matter; CP, crude protein; EE, ether extract; CF, crude fiber; NDF, neutral detergent fiber; ADF, acid detergent fiber; TP, Total Phosphorus; and GE, gross energy.

**Table 2:** Composition and nutrient levels of experimental diets (air-dry basis, %).

Items	CON	ZBL
Ingredients		
Alfalfa meal	32.00	32.00
Wheat bran	22.00	17.00
Corn	19.00	19.00
*Zanthoxylum bungeanum* leaf	0.00	5.00
Soybean meal	10.00	10.00
Wheat middling	3.00	3.00
Peanut vine	11.00	11.00
NaCl	0.40	0.40
CaHPO_4_	1.00	1.00
Limestone	0.60	0.60
Premix ^1^	1.00	1.00
Total	100.00	100.00
Nutritional level ^2^		
DE/(MJ/kg)	11.42	11.34
CP	16.38	16.42
CF	14.36	14.87
NDF	31.29	30.59
ADF	17.62	17.97
Lys	0.85	0.87
Met + Cys	0.61	0.59
Ca	0.79	0.83
TP	0.49	0.52

^1^ The premix provided the following per kg of diets: Fe 50 mg, Cu 5 mg, Zn 50 mg, Mn 8 mg, Se 0.1 mg, I 0.5 mg, VA 6000 IU, VD 1000 IU, VE 50 mg, VK 1 mg, VB_1_ 1 mg, VB_2_ 3 mg, VB_6_ 1.2 mg, VB_12_ 10 mg, nicotinic acid 30 mg, pantothenic acid 10 mg, choline 100 mg. ^2^ Amino acids and DE were calculated values, while the others were measured values.

**Table 3:** Primers used in this study.

Gene	Sequence (5′->3′)	
*MTHFD1L*	Forward primer	GCTGTTATTCAGGCAGGGGA
	Reverse primer	ACCTGAAGAGAAAGCCCGTG
*KY*	Forward primer	CCTATGACAACCAAGGGACAC
	Reverse primer	TAGGCGTGTGCATCTTTCCC
*PDZRN3*	Forward primer	CCTGTAAGGGGGCTGCTTTT
	Reverse primer	AGTCTTTGCCGTTGACCTGA
*PNPLA8*	Forward primer	CCCATGTGTCCTAAGGTAGCTG
	Reverse primer	ATGGCCTGCCACATCTTGTA
*TIMP1*	Forward primer	TGCAACTCCGACCTTGTCAT
	Reverse primer	GGGATTTGTGGGAGTACCCG
*KLF10*	Forward primer	CTCGCAGGCAGTCAGCTC
	Reverse primer	ATCTCCATTCTCTCCGGGGC
*SIX2*	Forward primer	GCCAAGGAAAGGTACGAGGAGA
	Reverse primer	CTGCCGTTCAGCGACGAAG
*GAPDH*	Forward primer	TCGGAGTGAACGGATTTGGC
	Reverse primer	TTCCCGTTCTCAGCCTTGAC

**Table 4:** Effects of *Zanthoxylum bungeanum* leaf on the growth performance of meat rabbits (*n* = 9 per group).

Items ^1^	CON	ZBL	*p*-Value
Initial weight (g)	1726.50 ± 4.73	1726.87 ± 4.62	0.956
Final weight (g)	2502.08 ± 32.07	2537.71 ± 22.39	0.378
ADG (g)	27.78 ± 1.03	28.97 ± 0.83	0.383
ADFI (g)	136.22 ± 2.19	138.97 ± 4.63	0.608
F/G	4.69 ± 0.12	4.92 ± 0.12	0.120

^1^ Abbreviations: ADG, average daily gain; ADFI, average daily feed intake; and F/G, feed-to-gain ratios.

**Table 5:** Effect of *Zanthoxylum bungeanum* leaf on slaughter performance and relative organ weight in meat rabbits (*n* = 9 per group).

Items	CON	ZBL	*p*-Value
Half-eviscerated yield percentage (%)	49.77 ± 0.63	51.01 ± 0.37	0.109
Eviscerated yield percentage (%)	48.11 ± 0.59	48.15 ± 0.37	0.956
Leg muscle rate (%)	25.66 ± 0.30	26.24 ± 0.37	0.242
Heart index (g/kg)	2.40 ± 0.06	2.42 ± 0.06	0.841
Liver index (g/kg)	26.00 ± 0.38	25.78 ± 0.35	0.679
Kidney index (g/kg)	5.35 ± 0.11	5.40 ± 0.12	0.774
Spleen index (g/kg)	0.57 ± 0.02	0.63 ± 0.04	0.172
Thymus index (g/kg)	1.66 ± 0.08	1.57 ± 0.09	0.445
Sacculus rotundus index (g/kg)	1.01 ± 0.01	0.97 ± 0.03	0.202

**Table 6:** The effect of *Zanthoxylum bungeanum* leaf on meat quality in the leg muscles of meat rabbits (*n* = 9 per group).

Items	CON	ZBL	*p*-Value
pH_45min_	6.71 ± 0.08	6.63 ± 0.09	0.500
pH_24h_	6.48 ± 0.03	6.49 ± 0.04	0.771
L*_45min_	41.97 ± 1.06	40.60 ± 0.80	0.318
a*_45min_	9.28 ± 1.27	8.48 ± 0.73	0.593
b*_45min_	1.94 ± 0.29	2.41 ± 0.37	0.333
L*_24h_	43.33 ± 1.08	42.21 ± 1.05	0.469
a*_24h_	15.25 ± 1.38	15.87 ± 1.41	0.758
b*_24h_	5.28 ± 0.45	4.85 ± 0.72	0.622
Drip loss (%)	1.01 ± 0.07	0.70 ± 0.07	0.006
Cooking loss (%)	31.53 ± 1.39	26.59 ± 1.18	0.015
Shear force (N)	76.69 ± 3.43	68.76 ± 5.03	0.211

**Table 7:** Effect of *Zanthoxylum bungeanum* leaf on antioxidant indices in leg muscle of meat rabbits (*n* = 9 per group).

Items ^1^	CON	ZBL	*p*-Value
T-AOC (U/mg prot)	0.16 ± 0.01	0.23 ± 0.02	0.010
GSH-PX (U/mg prot)	479.46 ± 21.31	569.57 ± 21.15	0.008
CAT (U/mg prot)	0.40 ± 0.02	0.45 ± 0.01	0.036
T-SOD (U/mg prot)	14.04 ± 0.71	16.87 ± 0.69	0.011
MDA (nmol/mg prot)	0.24 ± 0.02	0.17 ± 0.01	0.027

^1^ Abbreviations: T-AOC, total antioxidant capacity; GSH-PX, glutathione peroxidase; CAT, catalase; T-SOD, total superoxide dismutase; and MDA, malondialdehyde.

**Table 8:** Effect of *Zanthoxylum bungeanum* leaf on the medium- and long-chain fatty acid profile in the leg muscle of meat rabbits (mg/100 g).

Items ^1^	CON	ZBL	*p*-Value
C12:0	0.120 ± 0.014	0.070 ± 0.008	0.028
C14:0	4.609 ± 0.699	2.484 ± 0.336	0.021
C14:1 n-5	0.284 ± 0.037	0.156 ± 0.021	0.010
C15:0	2.101 ± 0.076	1.712 ± 0.144	0.037
C16:0	145.9 ± 5.292	122.2 ± 4.987	0.006
C16:1 n-7	8.272 ± 1.486	4.350 ± 0.716	0.032
C17:0	3.254 ± 0.142	2.844 ± 0.246	0.177
C18:0	67.95 ± 2.164	62.84 ± 3.404	0.225
C18:1 n-9 cis	82.81 ± 6.459	64.35 ± 5.207	0.043
C18:1 cis-11	8.415 ± 0.664	8.161 ± 0.395	0.747
C18:2 n-6	140.0 ± 5.019	125.4 ± 9.459	0.201
C18:3 n-6	0.416 ± 0.043	0.273 ± 0.016	0.013
C18:3 n-3	3.396 ± 0.283	2.256 ± 0.260	0.010
C20:0	0.125 ± 0.007	0.097 ± 0.008	0.018
C20:1 n-9	0.575 ± 0.071	0.466 ± 0.036	0.197
C20:2 n-6	2.930 ± 0.231	2.663 ± 0.223	0.418
C20:3 n-6	4.412 ± 0.203	4.263 ± 0.324	0.703
C20:4 n-6	59.20 ± 2.421	53.78 ± 3.292	0.206
C20:3 n-3	0.578 ± 0.072	0.477 ± 0.035	0.226
C20:5 n-3	0.837 ± 0.047	0.753 ± 0.028	0.150
C22:0	0.175 ± 0.014	0.147 ± 0.018	0.130
C22:4 n-6	10.73 ± 0.731	10.92 ± 1.067	0.881
C22:5 n-6	5.361 ± 0.371	5.216 ± 0.519	0.823
C24:0	0.284 ± 0.015	0.259 ± 0.025	0.392
C22:5 n-3	7.898 ± 0.465	7.246 ± 0.592	0.400
C22:6 n-3	1.096 ± 0.110	1.072 ± 0.089	0.866
SFA	224.7 ± 5.720	192.8 ± 8.550	0.008
UFA	337.4 ± 6.613	292.0 ± 15.07	0.021
MUFA	100.6 ± 8.373	77.66 ± 5.950	0.043
PUFA	236.8 ± 8.398	214.3 ± 13.70	0.183
Σn-3	19.17 ± 0.715	17.02 ± 1.147	0.134
Σn-6	223.0 ± 8.081	202.5 ± 13.15	0.205
Σn-6/Σn-3	11.65 ± 0.158	11.95 ± 0.466	0.542
AI	0.487 ± 0.020	0.455 ± 0.012	0.279
TI	0.997 ± 0.023	0.985 ± 0.016	0.682

^1^ SFA: saturated fatty acids; UFA: unsaturated fatty acids; MUFA: monounsaturated fatty acids; PUFA: polyunsaturated fatty acids; Σn-3: total n-3 polyunsaturated fatty acids; Σn-6: total n-6 polyunsaturated fatty acids; Σn-6/Σn-3: ratio reflecting the dietary balance between two classes of essential fatty acids; AI: atherogenic index; TI: thrombogenic index. AI = [C12:0 + (4 × C14:0) + C16:0]/ΣUFA; TI = (C14:0 + C16:0 + C18:0)/[(0.5 × ΣMUFA) + (0.5 × Σn-6) + (3 × Σn-3) + (Σn-3/Σn-6)]. Fatty acid concentrations are expressed as mg/100 g muscle (*n* = 9 per group). Trace components below 0.1 mg/100 g have been excluded.

## Data Availability

The data presented in this study have been deposited in the NCBI Sequence Read Archive (SRA) under BioProject accession numbers PRJNA1467413 (for microbiome data) and PRJNA1467479 (for transcriptomic data). Additional data are available from the corresponding author upon reasonable request.
